# Electrifying microbes for the production of chemicals

**DOI:** 10.3389/fmicb.2015.00201

**Published:** 2015-03-11

**Authors:** Pier-Luc Tremblay, Tian Zhang

**Affiliations:** Novo Nordisk Foundation Center for Biosustainability, Technical University of Denmark, HørsholmDenmark

**Keywords:** microbial electrosynthesis, bioelectrochemical systems, electricity, CO_2_ reduction, electron transfer mechanisms

## Abstract

Powering microbes with electrical energy to produce valuable chemicals such as biofuels has recently gained traction as a biosustainable strategy to reduce our dependence on oil. Microbial electrosynthesis (MES) is one of the bioelectrochemical approaches developed in the last decade that could have critical impact on the current methods of chemical synthesis. MES is a process in which electroautotrophic microbes use electrical current as electron source to reduce CO_2_ to multicarbon organics. Electricity necessary for MES can be harvested from renewable resources such as solar energy, wind turbine, or wastewater treatment processes. The net outcome is that renewable energy is stored in the covalent bonds of organic compounds synthesized from greenhouse gas. This review will discuss the future of MES and the challenges that lie ahead for its development into a mature technology.

## Introduction

**FIGURE 1:**
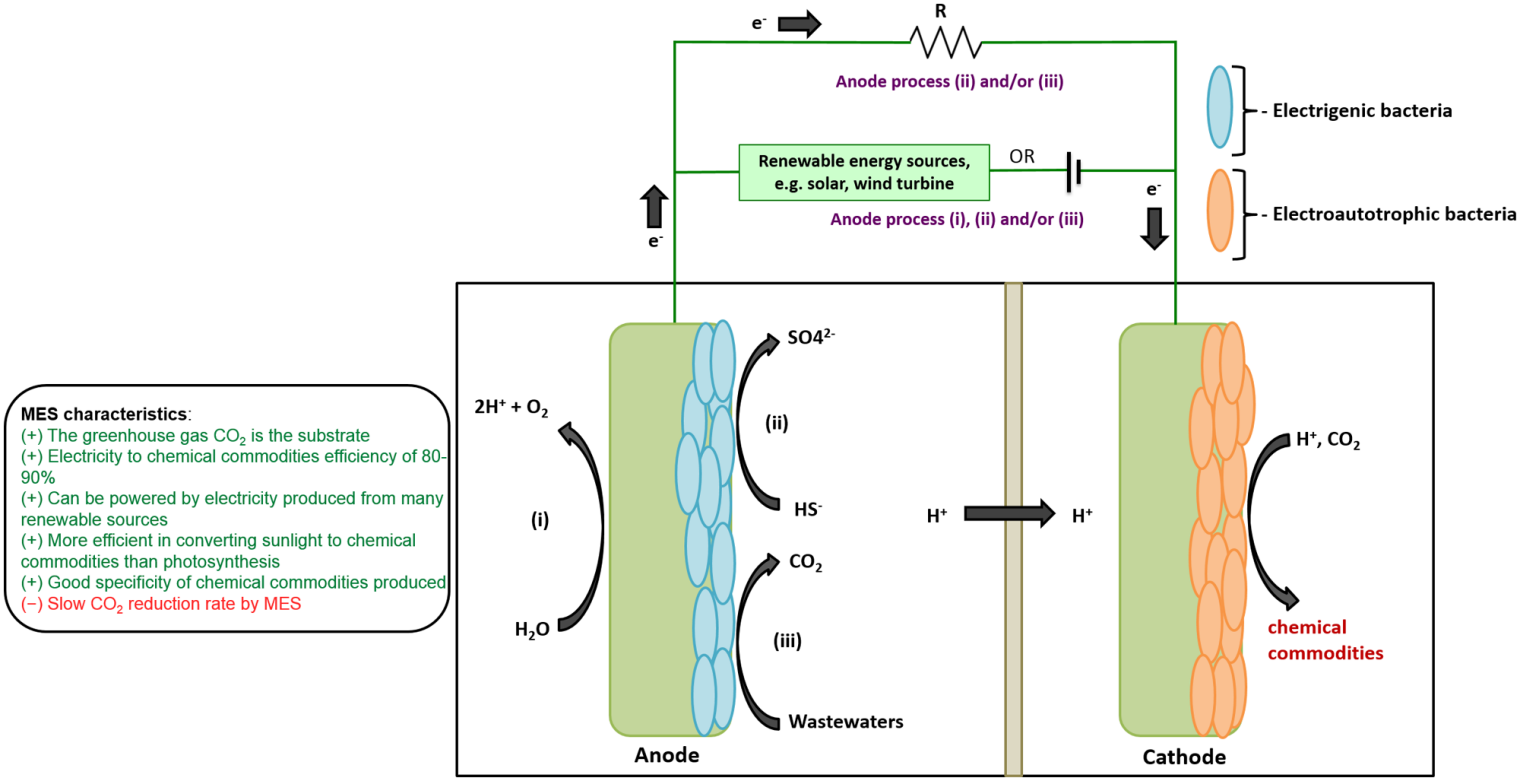
**Principle and flexibility of MES.** (i) MES can be coupled with different renewable energy sources such as wind and solar to produce a wide range of chemical commodities. MES can also be coupled to environment-friendly anodic processes such as (ii) sulfide oxidation and (iii) wastewaters treatment.

Microbial electrosynthesis (MES) happens when a microbial catalyst reduces CO_2_ into multicarbon chemical commodities with electrons derived from the cathode of a bioelectrochemical system designed primarily to perform biological reductive reactions (rBES; Rabaey and Rozendal, 2010; Rabaey et al., 2011; Lovley, 2012; Lovley and Nevin, 2013; Wang and Ren, 2013; Hallenbeck et al., 2014; Rosenbaum and Franks, 2014; **Figure 1**). rBES-driven processes also include electrofermentation, electrorespiration, and electromethanogenesis. Electrofermentation occurs when electrons coming from a cathode are supplied to a fermentative microbial catalyst shifting the fermentation balance toward the production of more reduced products (Rabaey and Rozendal, 2010; Kracke and Krömer, 2014). In the case of electrorespiration, a terminal electron acceptor such as fumarate is reduced by a respiratory microbial catalyst with electrons coming from a cathode (Park et al., 1999; Rabaey and Rozendal, 2010). Electromethanogenesis has similarities with MES since CO_2_ is the feedstock, but in this case CO_2_ will be reduced to methane by a methanogenic microbial catalyst using electrons derived from a cathode (Cheng et al., 2009; Villano et al., 2010; Kobayashi et al., 2013).

Besides its capacity of using CO_2_ directly as feedstock, the two other main qualities of MES are its energetic efficiency and its versatility (**Figure 1**). The electricity efficiency to chemical commodities of MES processes is ca. 80–90% (Nevin et al., 2010, 2011; Nie et al., 2013; Zhang et al., 2013). Many crop plants have sunlight efficiency to biomass below 3% (MacDonald, 2003), whereas common silicon solar cells are at least six times more efficient at capturing the sun energy (Green et al., 2014). Therefore, powering MES with electricity from solar cells could be a more potent strategy for storing the sun energy into the chemical bonds of multicarbon compounds (Nevin et al., 2010; Lovley and Nevin, 2011).

Microbial electrosynthesis is a versatile technology because the necessary electricity can be generated from multiple renewable sources. Apart from sun energy, MES can be powered with electricity produced by wind turbine. Renewable electricity sources are intermittent by nature and do not harmonize well with the market demand (Jürgensen et al., 2014). In this context, MES becomes a perfect technological fit making possible the direct storage of electricity surplus into value-added chemical commodities (Lovley and Nevin, 2011).

Microbial electrosynthesis can also be coupled with a bioelectrochemical system performing biological oxidation reactions (oBES). Processes driven by oBES are defined by the transfer of electrons from a microbial catalyst metabolizing a given substrate to an electrode collecting electricity. oBESs have been developed for a multitude of applications including wastewater treatments, *in situ* bioremediation, water desalination, biosensors, electrohydrogenesis, and various types of microbial fuel cells (Cheng and Logan, 2007; Cao et al., 2009; Lovley, 2009, 2012; Logan, 2010; Logan and Rabaey, 2012). Recent studies have shown that electrons coming from oBES-driven processes can be used to supply MES (**Figure 1**). In sulfide-driven MES, the electricity required for MES is generated by the abiotic oxidation of the toxic contaminant sulfide to sulfur and the subsequent biological oxidation of sulfur to sulfate (Gong et al., 2013). A similar process has also been developed to supply electrons for the electroproduction of methane from CO_2_ (Jiang et al., 2014). Moreover, MES and electromethanogenesis have been conducted in parallel with the biorecovery of cobalt at the cathode further illustrating the versatility of this technology (Huang et al., 2014).

A vibrant illustration of the significant progress made by MES in a relatively short period of time is that multicarbon compounds production rates by MES have been increased substantially over the last 4 years. For instance, the acetate production rate has been increased 433-fold from ca. 30 mM d^-1^ m^-2^ to ca. 1.3 mM d^-1^ cm^-2^, whereas the electron transfer rate was enhanced 521-fold from ca. 71 mA m^-2^ to ca. 3.7 mA cm^-2^ (Nevin et al., 2010; Marshall et al., 2012, 2013; Nie et al., 2013; Zhang et al., 2013; Jourdin et al., 2014; LaBelle et al., 2014; **Table 1**). However, the main obstacle for the development of MES as an economically viable technology is still the relatively slow microbial reduction rate of CO_2_ to multicarbon compounds in scalable rBES reactors. This review will discuss the ongoing efforts to increase MES productivity, stability, long-term efficiency, and versatility by optimizing microbial catalysts and electrochemical hardware and by characterizing the electron transfer mechanisms from cathode to microbe.

**Table 1:** Microbial electrosynthesis systems in chronological order of publication.

Microbial catalyst	Cathode	Comments	Reference
*Acidithiobacillus ferrooxidans*	-Platinum	-Fe(II)-mediated	Kinsel and Umbreit (1964)
*A. ferrooxidans*	-Platinum mesh	-Fe(II)-mediated	Nakasono et al. (1997)
*A. ferrooxidans*	-Platinum mesh -0.0 V (vs. Ag/AgCl)	-Fe(II)-mediated	Matsumoto et al. (1999)
*Leptospirillum ferrooxidans*	-Platinum mesh - +0.1 V (vs. Ag/AgCl)	-Fe(II)-mediated	Matsumoto et al. (2000)
*Sporomusa ovata*	-Graphite stick - –0.4 V (vs. SHE)	-Direct electron transfer -Acetate and 2-oxobutyrate produced	Nevin et al. (2010)
*A. ferrooxidans*	-Graphite felt - –0.0 V (vs. SCE)	-Direct electron transfer -Current density: 5 A m^-2^	Carbajosa et al. (2010)
*Clostridium aceticum* *Clostridium ljungdahlii* *Moorella thermoacetica* *Sporomusa silvacetica* *Sporomusa sphaeroides*	-Graphite stick - –0.4 V (vs. SHE)	-Direct electron transfer -Acetate, 2-oxobutyrate and formate produced	Nevin et al. (2011)
*Ralstonia eutropha*	-Indium foil - –1.6 V (vs. Ag/AgCl)	-Formate-mediated -Biofuels produced	Li et al. (2012)
Mixed community	-Graphite fiber brush/carbon rod/graphite plate - –0.439 V or –0.539 V (vs. SHE)	-Current density: 52 mA m^-2^ (Graphite plate) -Power density: 83 mWm^-2^ (graphite plate)	Pisciotta et al. (2012)
Mixed community	-Graphite granule - –0.59 V (vs. SHE)	-Acetate, methane, and H_2_ produced -Acetate production: >4 mM d^-1^	Marshall et al. (2012)
*Nitrosomonas europaea*	-Nickel, glassy carbon, or copper -Multiple potentials	-Ammonia-mediated -Multi-reactors system	Khunjar et al. (2012)
*Geobacter sulfurreducens*	-Stainless steel - –0.6 V (vs. Ag/AgCl)	-Direct electron transfer -Current density: 30 A m^-2^	Soussan et al. (2013)
*S. ovata*	-Modified carbon cloth - –0.6 V (vs. Ag/AgCl)	-Direct electron transfer -Best cathode modification: chitosan -Current density: 475 mA m^-2^ -Acetate production: 229 mM d^-1^ m^-2^	Zhang et al. (2013)
*S. ovata*	-Graphite plate	-Powered by sulfide/sulfur bioanode (0.3 V vs. SHE) -Acetate production: 49.9 mmol d^-1^ m^-2^	Gong et al. (2013)
*Mariprofundus ferrooxydans*	-Graphite —0.076 V (vs. SHE)	-Direct electron transfer -Cell-normalized electrode oxidation rate: 0.075 pmol electrons cell^-1^ h^-1^	Summers et al. (2013)
Mixed community	-Carbon felt - –1.15 V (vs. Ag/AgCl)	-Methane and acetate produced -Acetate production: 94.73 mg d^-1^	Jiang et al. (2013)
Mixed community	-Graphite granule - –0.59 V (vs. SHE)	-Acetate, H_2_, formate, butyrate, and propionate produced -Acetate production: 17.25 mM d^-1^	Marshall et al. (2013)
*S. ovata*	-Nickel nanowires anchored to graphite - –0.6 V (vs. Ag/AgCl)	-Direct electron transfer -Acetate production: 282 mM d^-1^ m^-2^	Nie et al. (2013)
Mixed community	-Carbon fiber rod - –0.4 V (vs. SHE)	-Direct electron transfer -Acetate, ethanol, 1-butanol, propionate, butyrate, and H_2_ produced -Current density: 34 mA m^-2^	Zaybak et al. (2013)
*Rhodopseudomonas palustris*	-Graphite rod -+0.1 V (vs. SHE)	-Light-driven -Current density: 1.5 μA cm^-2^	Bose et al. (2014)
Mixed community	-Graphite felt	-Cobalt reduction -Methane and acetate produced	Huang et al. (2014)
Mixed community	-Graphite plate modified with NanoWeb-RVC - –0.85 V (vs. SHE)	-Current density: 3.7 mA cm^-2^ -Acetate production: 1.3 mM d^-1^ cm^-2^	Jourdin et al. (2014)
*R. palustris*	-Carbon cloth - –0.22 V (vs. Ag/AgCl)	-Fe(II)-mediated -Light-driven -Multi-reactor system -Current density: 7.2 μA ml^-1^	Doud and Angenent (2014)
Mixed community	-Graphite granule - –0.6 to –0.8 V (vs. SHE)	-Acidic pH -Acetate, H_2_, and formate produced -Acetate production: 51.6 mM d^-1^ (–0.8 V) -High H_2_ production	LaBelle et al. (2014)

## The Microbial Catalysts

### Mixed Communities

Microbial electrosynthesis can be driven by two major types of microbial catalysts: mixed communities and pure cultures. In the case of mixed communities, the cathodic chamber of the MES system is inoculated with samples from wastewater, sludge, or sediment (**Table 1**). One of the main advantages of employing a mixed community for MES is that it eliminates the need to work under stringent sterile conditions required with pure culture-driven bioprocesses. Moreover, the MES system with the highest reported acetate production rate to date of 1.3 mM d^-1^ cm^-2^ was driven by an uncharacterized mixed community (Jourdin et al., 2014). Mixed community-driven MES systems described until now mainly produce acetate because the microbial population quickly become dominated by acetogenic bacteria like *Acetobacterium* sp. (Marshall et al., 2012, 2013; LaBelle et al., 2014) and *Eubacterium* sp. (Pisciotta et al., 2012). There is also simultaneous production of methane due to the coexistent methanogens, unless an inhibitor of methanogenesis is added to the cathode reactor (Marshall et al., 2012, 2013; Pisciotta et al., 2012; Jiang et al., 2013; Huang et al., 2014). Hydrogen and formate produced biologically or abiotically at low cathode potential are other compounds frequently found in mixed community-driven MES reactors (Marshall et al., 2012, 2013; Pisciotta et al., 2012; Zaybak et al., 2013; LaBelle et al., 2014). Furthermore, in a study for the development of a method to facilitate the start-up of autotrophic biocathodes in rBESs, the mixed community microbial catalysts were reported to produce at least six products: butanol, ethanol, hydrogen, acetate, propionate, and butyrate (Zaybak et al., 2013). This study gives a good example of the difficulty generating a single specific product when employing mixed communities to drive MES processes. Other than compromising the purity of the desired product, it also complicates the separation process and reduces the electricity conversion efficiency to a specific multicarbon compound.

### Pure Cultures

#### Acetogenic Bacteria

Diverse autotrophic pure cultures have been employed successfully in the role of microbial catalysts for MES systems. As illustrated by several studies on mixed community-driven MES, acetogens reducing CO_2_ through the Wood–Ljungdahl pathway (Drake et al., 1997, 2008; Ragsdale and Pierce, 2008) are dominating and efficient electroautotrophs. Pure cultures of Gram negative acetogens like *Sporomusa silvacetica* and *Sporomusa sphaeroides* and Gram positive acetogens like *Clostridium ljungdahlii*, *Clostridium aceticum* and the thermophile *Moorella thermoacetica* are all capable of reducing CO_2_ to multicarbon compounds by MES (Nevin et al., 2011). Among all the tested acetogenic bacteria, *Sporomusa ovata* DSM-2662 was the most efficient electroautotroph with acetate production rates as high as 282 mM d^-1^ m^-2^ and with electricity conversion efficiency to acetate typically above 80% (Nevin et al., 2010; Gong et al., 2013; Nie et al., 2013; Zhang et al., 2013). The production of negligible amount of 2-oxobutyrate and formate in comparison to acetate by *S. ovata* was also reported (Nevin et al., 2010, 2011). Acetogens like *S. ovata* have high electricity conversion efficiency to chemical commodities compared to autotrophic bacteria with other types of carbon fixation metabolisms because CO_2_ is the sole electron acceptor during acetogenesis (Drake et al., 1997, 2008; Ragsdale and Pierce, 2008). Thus, most of the electrons derived from the cathode will end up in reduced multicarbon products. Moreover, a study by Fast and Papousakis (2012) established that the Wood–Ljungdahl pathway is the most energetically efficient non-photosynthetic carbon fixation pathway for the electroproduction of acetate and ethanol (Fast and Papousakis, 2012).

#### Autotrophic Fe(II) Oxidizing Bacteria

Autotrophic Fe(II) oxidizing bacteria are also capable of reducing CO_2_ by MES. The acidophilic aerobic Fe(II) oxidizer *Acidithiobacillus ferrooxidans* (Kinsel and Umbreit, 1964; Nakasono et al., 1997; Matsumoto et al., 1999) and *Leptospirillum ferrooxidans* (Matsumoto et al., 2000) were able to grow in a bioelectrochemical system by using electrons coming from electrochemically reduced Fe(II). Moreover, *A. ferrooxidans* (Carbajosa et al., 2010) and the neutrophilic aerobic Fe(II) oxidizer *Mariprofundus ferrooxydans* (Summers et al., 2013) were shown to draw current directly from a poised cathode in the absence of a redox mediator to grow with CO_2_ as the source of carbon. The current draw was 5 A m^-2^ with *A. ferrooxidans* (Carbajosa et al., 2010) whereas the cell-normalized electrode oxidation rate was 0.075 pmol electrons cell^-1^ h^-1^ with *M. ferrooxydans* (Summers et al., 2013). MES systems driven by these Fe(II) oxidizing bacteria were poised at potential closer to 0 V vs. SHE compared to all the other reported MES systems (**Table 1**). Potential closer to 0 V at the cathode could translate in lower energy requirements (Lovley and Nevin, 2013). However, in those systems O_2_ is the final electron acceptor which means that a significant amount of electrons will not be used to reduce CO_2_ into multicarbon commodities but to reduce O_2_ and to generate biomass.

#### Ammonia-Oxidizing Bacteria

Ammonia can be employed as a redox mediator in MES system to promote the growth of ammonia-oxidizing bacteria (Khunjar et al., 2012; **Table 1**). In the study by Khunjar et al. (2012), nitrite was reduced electrochemically to ammonia in the first reactor and then was fed to a second reactor containing the ammonia-oxidizing bacteria *Nitrosomonas europaea*. Electrons from ammonia were used by *N. europaea* to produce biomass from CO_2_ and to generate nitrite that was then recycled in the first electrochemical reactor. However, like the MES system driven by autotrophic Fe(II)-oxidizer, *N. europaea* requires O_2_ as an electron acceptor and thus this system has the same efficiency issue.

#### Recombinant Microbial Catalyst

The only example to date of a MES process driven by a genetically engineered microbial catalyst was a recombinant strain of the chemolithotrophic bacterium *Ralstonia eutropha* developed for the electroproduction of biofuels (Li et al., 2012). The recombinant strain of *R. eutropha* generated 140 mg/ml of biofuels over a period of ca. 100 h by oxidizing formate that was electrochemically produced at the surface of a poised cathode. This MES process required O_2_ as the final electron acceptor and a cathode poised at very low potential of –1.6 V vs. Ag/AgCl to generate the necessary electron shuttle formate. These two factors are expected to significantly lower the overall efficiency of this MES system due to the energy loss for O_2_ reduction and formate production. Nevertheless, this study demonstrates that one of the advantages of using a pure culture for MES is that it can be genetically engineered to optimize the metabolism of the microbial catalyst and to increase the range of possible products.

#### Geobacter sulfurreducens

Evidence suggest that *Geobacter sulfurreducens* can also reduce CO_2_ into a multicarbon compound by MES (Soussan et al., 2013; **Table 1**). *G. sulfurreducens* is a well-characterized electrigenic bacterium generating power densities in oBESs as high as 3.9 W/m^2^ (Yi et al., 2009). *G. sulfurreducens* pre-grown with acetate as an electron donor and carbon source was also shown to have the capacity to accept electrons from a cathode to reduce fumarate (Gregory et al., 2004; Dumas et al., 2008) or uranium(VI; Gregory and Lovley, 2005) after the depletion of acetate. In a study by Soussan et al. (2013), *G. sulfurreducens* first used electrons derived from the cathode to reduce the final electron acceptor fumarate into succinate. When all the fumarate was depleted, *G. sulfurreducens* appeared to start producing glycerol by a process combining CO_2_ possibly reduced electrochemically to bicarbonate with succinate. Interestingly, a previous report demonstrated that *G. sulfurreducens* cannot grow with CO_2_ alone (Coppi et al., 2004). Recently, the closely related species *Geobacter metallireducens* was shown to be capable to grow autotrophically with formate as the sole source of carbon (Feist et al., 2014). *G. metallireducens* genome encodes two known carbon fixation pathways, the reductive TCA cycle and the dicarboxylate–hydroxybutyrate cycle, both of which are not present in the genome of *G. sulfurreducens*. It has been suggested that formate could be assimilated by *G. sulfurreducens* in the presence of a small quantity of acetate via the pyruvate formate lyase (Speers and Reguera, 2012). However, in Soussan et al. (2013) study, the cathode is poised at a potential (–0.6 V vs. Ag/AgCl) too high for CO_2_ reduction to formate, hence the biochemical mechanism for the assimilation of CO_2_/bicarbonate and the production of glycerol remains unclear.

#### Photosynthetic Fe(II)-Oxidizing Bacteria

Microbial electrosynthesis system can also be exposed to light to provide energy for CO_2_ reduction by anaerobic photosynthetic Fe(II)-oxidizing bacteria. *Rhodopseudomonas palustris* is the microbial catalyst in two reported photobiocathode-based MES studies. In the first study, *R. palustris* was accepting electrons from a poised cathode in a one-reactor system to reduce CO_2_ with an electron transfer rate of 1.5 μA cm^-2^ (Bose et al., 2014). Electron uptake was stimulated by light exposure, but occurred at a significantly lower rate in the dark. However, it is not clear if *R. palustris* was accepting electrons directly from the cathode or from Fe(II) reduced electrochemically. The second study described a multi-reactor system similar to the aforementioned ammonia shuttling MES system by Khunjar et al. (2012). The redox mediator Fe(III) was first reduced abiotically by a cathode to Fe(II) in an electrochemical reactor before being fed to a second photobioreactor where *R. palustris* was growing with CO_2_ as the sole carbon source, Fe(II) as the electron source, and light (Doud and Angenent, 2014).

## Electron Transfer from the Cathode to the Microbial Catalyst

### Indirect Electron Transfer

#### Exogenous Electron Shuttles

**FIGURE 2:**
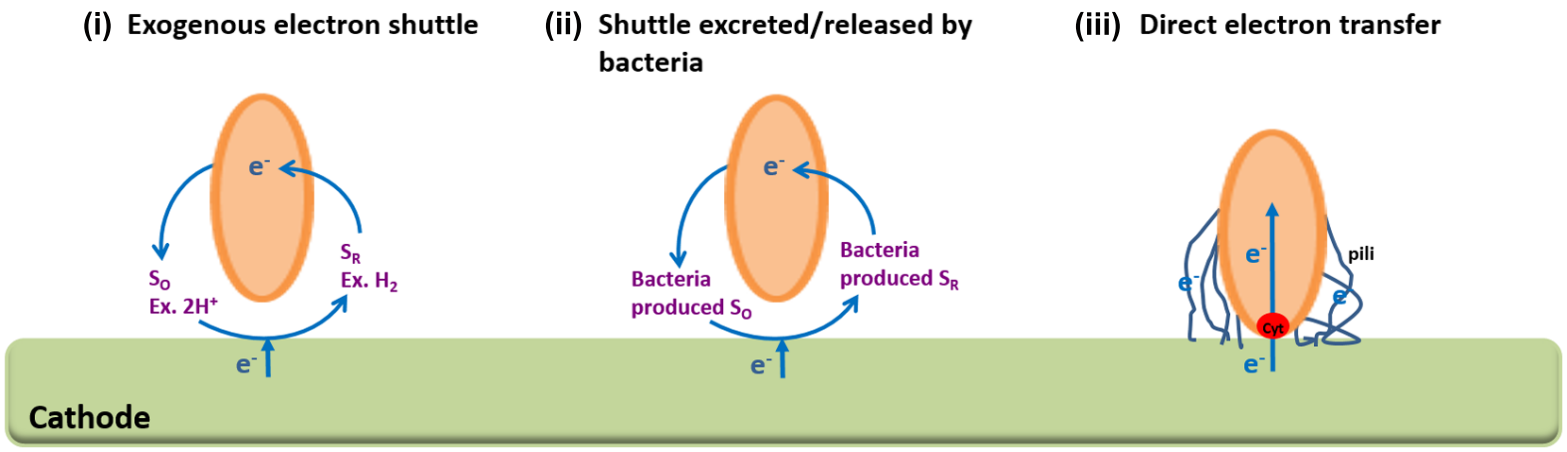
**Possible extracellular electron transfer mechanisms from the cathode to the microbial catalyst.** Indirect electron transfer via **(i)** an exogenous shuttle or **(ii)** a shuttle released/excreted by the microbial catalyst. **(iii)** Direct electron transfer via bacterial outer surface components such as c-type cytochromes or pili. SO is oxidized electron shuttle and SR is reduced electron shuttle.

Understanding the electron transfer mechanisms involved in MES could lead to major breakthroughs in the effort to increase the electron transfer rate between the cathode and the microbial catalyst. Electrons can either be transferred directly or indirectly via a shuttle (Patil et al., 2012; **Figure 2**). H_2_, formate, Fe(II) and ammonia have all been reported to function as redox mediator in MES systems (Lovley and Nevin, 2013). H_2_ has been the most prevalent (**Table 1**) since it requires only that the cathode in the MES reactor is poised at a lower potential than –0.41 V (vs. SHE). Under this condition, significant quantities of H_2_ are generated from the electrons coming from the cathode and the protons migrating from the anodic chamber. However, employing H_2_ as a redox mediator for MES is not optimal because its low solubility might cause energy losses.

#### Shuttles Excreted/Released by Bacteria

Another possible indirect electron transfer mechanism is that microbial catalysts could be producing and excreting their own soluble redox mediators to carry electrons from the cathode. Redox mediators either excreted by the bacteria such as phenazine and riboflavin or released after cell death such as vitamin B12 or DNA have all been suggested as possible components involved in extracellular electron transfer (Rosenbaum et al., 2011).

### Direct Electron Transfer

Direct electron transfer requires physical contacts between extracellular components of the microbial catalyst involved in the transport of electrons and the cathode. Direct electron transfer from the cathode to the microbial catalyst in the absence of a redox mediator has been shown to occur in MES processes driven by the following electroautotrophic species: *A. ferrooxidans* (Carbajosa et al., 2010; Rodrigues and Rosenbaum, 2014), *M. ferrooxydans* (Summers et al., 2013) and a group of acetogenic bacteria (Nevin et al., 2010, 2011; Nie et al., 2013; Zhang et al., 2013). This conclusion is mainly based on the fact that all these MES systems were operated with cathodes poised at potentials too high to produce significant quantity of H_2_. Moreover, driving MES systems with cathodes poised at lower potentials than the formal potential of the 2H^+^/H_2_ couple does not exclude the possibility of direct electron transfer. A study by Marshall et al. (2012) reported evidence that direct electron transfer was occurring at the same time as hydrogen-mediated electron transfer. This suggests that microbial catalysts are capable of acquiring electrons from a cathode through many paths simultaneously.

#### Electromethanogenic Bacteria

Recently, it was demonstrated that a mutant strain of *Methanococcus maripaludis* lacking all its catabolic hydrogenases was still capable of performing electromethanogenesis (Lohner et al., 2014) thus providing supplementary significant evidence that electrotrophic bacteria can accept electrons directly from the cathode independently of the redox mediator H_2_.

#### The Model Metal-Reducing Bacteria

Two of the most detailed studies about electron transfer mechanisms from the cathode to the microbe have been done with the two model metal-reducing bacteria *G. sulfurreducens* (Strycharz et al., 2011) and *Shewanella oneidensis* (Ross et al., 2011). In *G. sulfurreducens*, the monoheme *c*-type cytochrome GSU3274 predicted to be localized in the periplasm was found to be specifically required for electron transfer from the cathode demonstrating the importance of *c*-type cytochromes for electron uptake. Like *G. sulfurreducens*, *S. oneidensis* can do electrorespiration accepting electrons from the cathode to reduce fumarate into succinate (Ross et al., 2011). Based on functional genetic studies, Ross et al. (2011) proposed a model where the respiratory pathway normally responsible for transferring electron from the cytoplasmic metabolism to extracellular electron acceptors (Shi et al., 2012) works in reverse. Therefore, the decaheme outer membrane *c*-type cytochrome MtrC, the decaheme periplasmic *c*-type cytochrome MtrA, the porin MtrB responsible for connecting MtrC to MtrA, the tetraheme cytoplasmic membrane-associated *c*-type cytochrome CymA and the menaquinone pool are all critical components of the electron uptake pathway of *S. oneidensis*.

#### Electroautotrophic Bacteria

Direct electron transfer pathways are poorly characterized in electroautotrophic bacteria. For *A. ferrooxidans*, experimental evidence indicated that a Fe species excreted by the cells in the cathode biofilm could be responsible for electron uptake (Carbajosa et al., 2010). It has been speculated that *c*-type cytochromes which are critical components for the uptake of electrons from extracellular Fe(II) could also be involved in the transport of electron from the cathode (Rosenbaum et al., 2011; Sydow et al., 2014). In support of this hypothesis, metagenomics and metaproteomics of the mixed community populating a self-regenerating biocathode suggest that a member of the *Chromatiaceae* family reduced CO_2_ with electrons acquired directly from the cathode via *c*-type cytochromes and other proteins associated with Fe(II) oxidation (Wang et al., 2015).

Much less information is known about how electrons are acquired by acetogens from the cathode. Recently, a genetic system developed for the Gram positive bacterium *C. ljungdahlii* (Leang et al., 2013; Banerjee et al., 2014; Ueki et al., 2014) led to the confirmation of the identity of the proton pump responsible for the generation of a proton motive force essential for growth with CO_2_ as the sole source of carbon (Tremblay et al., 2013). This study provided insights on the energy conservation mechanism involved in the electroautotrophic growth of acetogens. The electron uptake mechanism of *C. ljungdahlii* is expected to be significantly different compared to other electrotrophic bacteria because it cannot synthesize *c*-type cytochromes or quinones (Köpke et al., 2010). The availability of a genetic toolbox should accelerate the characterization of the specificities of *C. ljungdahlii*’s electron transfer pathway and could provide general information about electron uptake by other Gram positive bacteria.

The genome sequence of the Gram negative and acetogenic species *S. ovata* has recently been made available (Poehlein et al., 2013). Genes coding for *c*-type cytochromes and type IV pili, two components of bacterial extracellular electron transfer mechanisms, are present in the genome. As mentioned before, *c*-type cytochromes are critical components of extracellular electron transfer pathways in both electrigenic and electrotrophic bacteria. In *Geobacter* spp., type IV pili are filaments with metallic-like conductivity facilitating long-range electron transfer (Lovley, 2011, 2012; Malvankar and Lovley, 2012; Vargas et al., 2013; Malvankar et al., 2014) involved in the reduction of insoluble electron acceptors (Reguera et al., 2005; Tremblay et al., 2012). Ubiquinone (Möller et al., 1984), another critical component of electron transport pathway, was also detected in *S. ovata* and genes coding for enzymes involved in its biosynthesis were found in the genome (Poehlein et al., 2013). *S. ovata* possesses several well-characterized components of microbial extracellular transfer pathways which indicates that electron uptake by *S. ovata* could have similarities with other electrigenic or electrotrophic bacteria. Recently, an acetogenic bacteria closely related to *S. sphaeroides* was shown to do acetogenesis with metallic iron (F(0)) as the sole electron donor suggesting that direct electron transfer could be a useful strategy for Gram negative acetogens in multiple environments (Kato et al., 2015).

## The Electrochemical Hardware

### Cathode Materials Tested for Current Draw

A lot of effort has been put into optimizing rBESs/MES by selecting or developing more efficient and less expensive components for bioelectrochemical reactors (Krieg et al., 2014). Until now, most of the studies published on this topic are presenting the impact of different types of biocompatible cathodes on the performance of rBESs/MES. Cathodes described in the literatures relied mainly on carbonaceous materials (**Table 1**). Indium foil (Li et al., 2012) and platinum (Kinsel and Umbreit, 1964; Nakasono et al., 1997; Matsumoto et al., 1999) have also been tried in rBESs but the most efficient cathode material to date for electron transfer is stainless steel with current draw as high as 30 A m^-2^ with *G. sulfurreducens* as the microbial catalyst (Soussan et al., 2013).

Conductive materials that will self-assemble in the cathodic biofilm are another approach to enhance electron transfer in rBESs. A *S. oneidensis* biofilm assembled with embodied graphene oxide could uptake electrons 74 times more efficiently (Yong et al., 2014). Oligoelectrolytes can also facilitate electron transfer from the cathode by inserting itself in the lipid membrane of bacteria to enable transmembrane charge transfer as demonstrated by Thomas et al. (2013).

### Cathode Materials Tested for Chemical Production

Cathodes employed for MES are generally made of carbonaceous materials like graphite. These basic cathodes can be treated or coated with other materials resulting in modifications of their surface.

#### Untreated Surface

When chemical commodities production rate is not normalized to total surface area of cathode, one of the best materials is granular graphite with acetate production from a mixed community catalyst reaching 51.6 mM per day or 3.0 gL^-1^ d^-1^ (LaBelle et al., 2014). As a packed structure, the main advantage of granular graphite over other carbonaceous cathodes is the high specific area for bacterial adhesion (Wei et al., 2011).

#### Treated Surface

Modifications of carbonaceous cathodes have resulted in critical improvements in MES systems driven either by mixed communities or by pure cultures. Recently, Jourdin et al. (2014) coated carbon nanotubes on reticulated vitreous carbon (NanoWeb-RVC) to enhance bacterial attachment in a mixed community-driven MES system resulting in the highest normalized current density and the highest normalized acetate production rate reported for any MES systems until now (**Table 1**). The authors suggested that this performance improvement was due to the high surface to volume ratio of NanoWeb-RVC responsible for enhanced bacterial adhesion and effective mass transfer within the electrode-biofílm superstructure (Jourdin et al., 2014). The higher reported acetate production rate and current density compared to other MES systems relying on direct electron transfer (Nevin et al., 2010, 2011; Nie et al., 2013; Zhang et al., 2013) can also be attributed in part to the low potential of the cathode (–0.85 V vs. SHE) promoting indirect electron transfer that could be advantageous for the unknown bacterial species populating the reactor. Maintaining the cathode at this potential is possibly responsible for the slightly lower electricity to acetate efficiency of 70% observed with this MES reactor. Although this system is promising, the volumetric acetate production rate of ca. 0.025 gL^-1^ d^-1^ is 120-fold lower than that of the MES system also driven by a mixed community developed by LaBelle et al. (2014). Scaling up test for the NanoWeb-RVC biocathode of Jourdin et al. (2014) will indicate its real potential for industrial applications.

A number of modified carbonaceous cathodes have been proposed for MES systems driven by the pure culture catalyst *S. ovata* with significant success. Functionalization of carbon cloth cathodes with chitosan or other compounds conferring a positive charge to the electrode surface with the aim of increasing interactions with negatively charged bacteria like *S. ovata* (Zhang et al., 2013), resulted in higher cell density at the cathode surface (up to 9-fold increase), in better electron transfer (up to 6.7-fold increase as 475 mA m^-2^) and in higher normalized acetate production rates (up to 7.6-fold increase as 229 mM d^-1^ m^-2^). In a second approach, carbon cloth cathodes were treated with metal, such as gold, palladium, or nickel nanoparticles to harness their exceptional catalytic activities for MES processes. Significant increases in normalized acetate production rate by MES were recorded with all three metals in the range of 100–200 mM d^-1^ m^-2^ (Zhang et al., 2013). Modifying polyester- or cotton-based textile composite cathodes with carbon nanotubes to create a three-dimensional matrix with more surface area available for bacteria also resulted in significant increase in the productivity of MES of ca. 100 mM d^-1^ m^-2^ (Zhang et al., 2013). In a later study by the same group, using nickel nanowires anchored to graphite electrode resulted in the highest normalized acetate production rate recorded for a pure culture-driven MES system due to enhanced surface area and the generation of a porous structure (Nie et al., 2013; **Table 1**).

## Concluding Remarks

Microbial electrosynthesis is a young technology that made significant progress in terms of productivity over the last 5 years. Large efforts have been done to optimize known microbial catalysts for MES and to screen new ones with strong electroautotrophic properties (Rodrigues and Rosenbaum, 2014). Meanwhile, cathodes fabricated with novel materials or designed with better spatial arrangement are being explored and developed. Optimization of other parts of the electrochemical hardware such as the ion-exchange membrane and the current-collecting structure for MES processes are also ongoing (Varcoe et al., 2014). For example, it has been suggested that anion-exchange membranes can be exploited in the *in situ* selective extraction of acetate produced by MES at a lower energetic cost (Andersen et al., 2014). Furthermore, understanding in detail how electrons are transferred from the cathode to the microbial catalyst will help devise better-designed strategies to improve all aspects of MES. If this rate of improvement is maintained in the future, MES could fulfill its promise as an energetically efficient, environment-friendly, and versatile bioproduction strategy.

## Conflict of Interest Statement

The authors declare that the research was conducted in the absence of any commercial or financial relationships that could be construed as a potential conflict of interest.
